# The impact of digital literacy on digital resilience among nursing interns: the mediating role of self-efficacy

**DOI:** 10.3389/fpubh.2026.1822817

**Published:** 2026-04-09

**Authors:** Jihong Wang, Jinyan Li, Meng Liu, Yuxiao Wang, Xichao Xia, Fengxia Wang

**Affiliations:** Pingdingshan University, Pingdingshan, China

**Keywords:** clinical education, digital literacy, digital resilience, mediation analysis, nursing interns, self-efficacy

## Abstract

**Objective:**

This study aimed to investigate the current status of digital resilience among nursing interns, analyze its relationship with digital literacy and self-efficacy, and examine the mediating role of self-efficacy between digital literacy and digital resilience.

**Methods:**

A cross-sectional study was conducted from August to November 2025 using convenience sampling. A total of 823 nursing interns from eight tertiary Grade A hospitals in China were surveyed using the Digital Resilience Scale for Undergraduate Nursing Students, the Medical Students’ Digital Literacy Scale, and the General Self-Efficacy Scale.

**Results:**

The total digital resilience score was 138.95 ± 13.45. Digital resilience was positively correlated with digital literacy and self-efficacy (*r* = 0.713 and 0.590, respectively, *p* < 0.01). Self-efficacy partially mediated the relationship between digital literacy and digital resilience, accounting for 19.10% of the total effect.

**Conclusion:**

Digital resilience among nursing interns is moderate with room for improvement. Digital literacy and self-efficacy are significant predictors of digital resilience, with self-efficacy playing a mediating role. These findings suggest that nursing education should integrate digital competency development with psychological empowerment strategies to enhance interns’ adaptability in digital healthcare environments.

## 1 Introduction

The integration of digital technologies such as artificial intelligence, virtual reality, and intelligent nursing systems into clinical practice is transforming healthcare delivery and nursing education (1, 2). Recent developments in 2024–2025 have accelerated this transformation, with generative AI, Internet of Medical Things (IoMT), and predictive analytics becoming integral to patient care (3). The COVID-19 pandemic served as a catalyst for digital health adoption, yet it also exposed significant gaps in healthcare professionals’ preparedness for digital environments (4). These technologies not only reshape clinical workflows but also create new learning environments for nursing students, particularly during clinical internships. As future healthcare professionals, nursing interns must develop the capability to adapt to and thrive in digitally mediated clinical settings—a capability conceptualized as digital resilience.

Digital resilience refers to an individual’s ability to effectively cope with, adapt to, and recover from challenges encountered in digital environments, including technical failures, information overload, and cybersecurity threats (5). Despite growing recognition of its importance, empirical research on digital resilience remains in its nascent stage, particularly within nursing populations. Existing studies have predominantly focused on general student populations (6) or practicing nurses (7), leaving a significant gap in understanding how nursing interns—who are simultaneously learners and emerging professionals—develop digital resilience during their critical transition from academic to clinical settings. For nursing interns, this resilience directly influences their clinical performance, patient safety outcomes, and professional development (7). Moreover, while digital resilience has been linked to various individual and contextual factors, the psychological mechanisms through which specific competencies translate into resilient behaviors remain poorly understood.

Digital literacy—encompassing the knowledge, skills, and attitudes required to effectively use digital technologies—serves as a foundational competency for navigating digital healthcare environments (8). Recent meta-analytic evidence suggests that digital literacy interventions can enhance professional competencies across healthcare disciplines (9); however, the pathway from literacy to resilient behavior is not direct. Bandura’s social cognitive theory (1977) posits that self-efficacy—the belief in one’s capability to execute behaviors—mediates the relationship between skills and outcomes, suggesting that digital literacy may influence resilience through confidence-building mechanisms. Previous research suggests that digital literacy may influence digital resilience (10), but this relationship has not been empirically tested in nursing education contexts, and the mediating mechanisms remain speculative.

Self-efficacy, defined as an individual’s belief in their capability to execute specific behaviors to achieve desired outcomes (11), has been identified as a critical psychological resource in technology adoption and stress coping. In healthcare education, self-efficacy predicts technology acceptance (12), simulation performance (13), and resilience during clinical transitions (14). However, few studies have examined whether self-efficacy serves as a bridge between digital competencies and adaptive outcomes. Qiu (15) found that self-efficacy mediated the relationship between digital literacy and employability among vocational students, suggesting potential generalizability to healthcare contexts. Nevertheless, the specific role of self-efficacy in linking digital literacy to digital resilience among nursing interns requires empirical verification.

**Figure 1:**
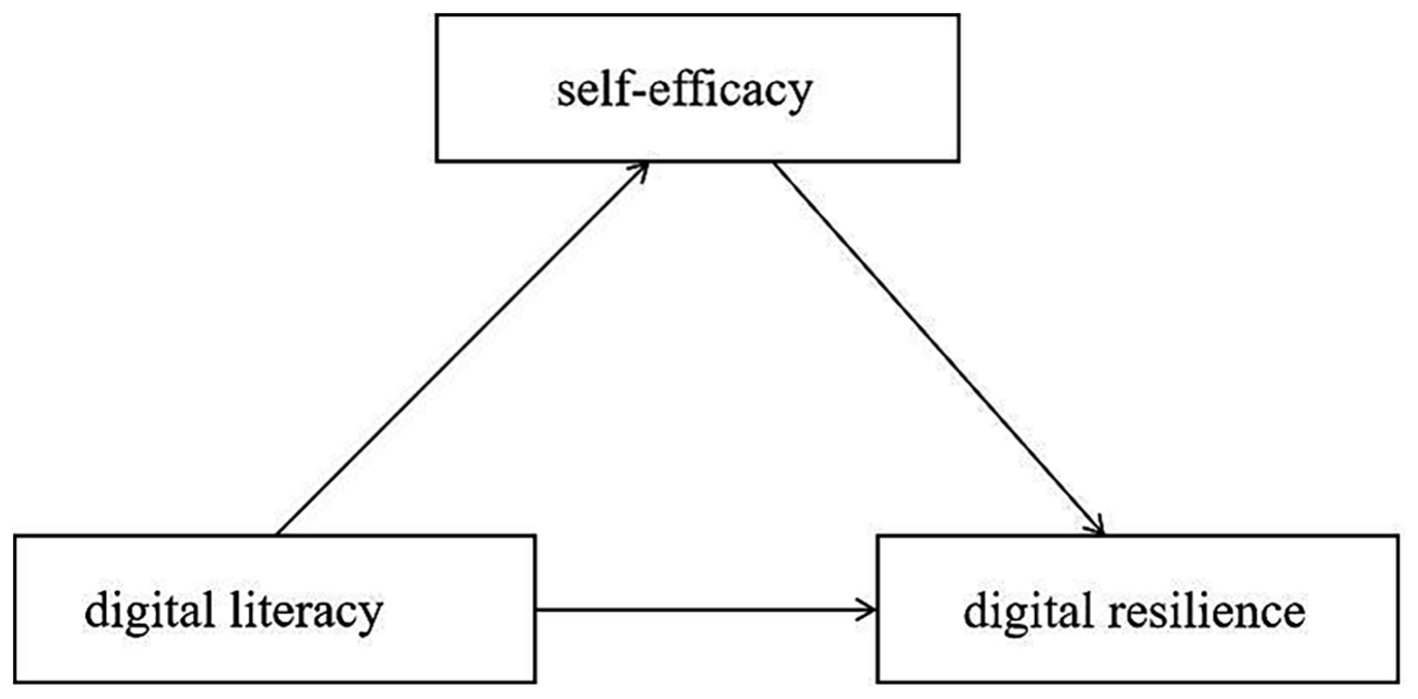
Conceptual framework and hypotheses.

Based on the theoretical framework of social cognitive theory (11) and the emerging empirical evidence on digital competencies in healthcare, this study proposes the following hypotheses (Figure 1):

*H1*: Digital literacy is positively associated with digital resilience among nursing interns.

*H2*: Self-efficacy is positively associated with digital resilience among nursing interns.

*H3*: Self-efficacy mediates the relationship between digital literacy and digital resilience.

These hypotheses are grounded in the theoretical proposition that digital skills enhance confidence, which in turn facilitates adaptive responses to digital challenges. By testing this mediation model, the study aims to elucidate the psychological mechanisms underlying digital resilience development and provide evidence for targeted educational interventions.

This study aims to address these research gaps by investigating: (1) the current status of digital resilience among nursing interns; (2) the relationships among digital literacy, self-efficacy, and digital resilience; and (3) whether self-efficacy mediates the relationship between digital literacy and digital resilience. The findings will provide empirical evidence to inform educational interventions aimed at enhancing nursing interns’ preparedness for digital healthcare environments.

## 2 Methods

### 2.1 Study design and participants

A cross-sectional study was conducted from August to November 2025 using convenience sampling, a method commonly employed in nursing research when comprehensive sampling frames are unavailable and when the research aims to explore associations rather than estimate population prevalence (16, 17). While convenience sampling limits generalizability, it facilitates access to specialized populations—in this case, nursing interns actively engaged in clinical rotations—where probability sampling is often impractical (18). To mitigate selection bias, we recruited from eight geographically dispersed tertiary hospitals across multiple provinces in China, ensuring diversity in institutional contexts.

Participants were recruited from eight tertiary Grade A hospitals in China. Inclusion criteria were: (1) full-time nursing students currently completing clinical internships; (2) at least 1 month of clinical experience with digital nursing tools; and (3) voluntary participation with signed informed consent. Exclusion criteria included: (1) non-nursing majors; (2) insufficient clinical experience with digital tools; and (3) cognitive or communication barriers preventing questionnaire completion. This study protocol has been approved by the Ethics Committee of Pingdingshan University (approval number: PDSU20250310). All participants provided informed consent and voluntarily participated in this study.

Sample size was estimated based on the rule of 10–15 participants per predictor variable for multiple regression analysis (19). A total of 20 predictor variables were considered, including demographic covariates (gender, place of origin, internship duration, digital tool use frequency, digital training participation) and main study variables (digital literacy, self-efficacy). This yielded a minimum required sample of 200–300 participants. Accounting for a 20% non-response rate, the target sample was set at 240–360 participants. The final sample of 823 participants exceeded this requirement, providing >90% statistical power to detect medium effect sizes (*f*^2^ = 0.15) at α = 0.05 (20). The larger sample also enhances the precision of parameter estimates and the stability of bootstrapped mediation analyses (21).

### 2.2 Measures

#### 2.2.1 General information questionnaire

This self-designed questionnaire collected demographic and clinical characteristics including gender, age, educational background, internship duration, frequency of digital tool use, and participation in digital skills training.

#### 2.2.2 Digital resilience scale for undergraduate nursing students

The 39-item scale developed by Li (22) was used, comprising five dimensions: digital risk perception, cognitive coping strategies, knowledge and skill acquisition, digital stress management, and digital environment adaptation. Items were rated on a 5-point Likert scale (1 = completely disagree to 5 = completely agree), with total scores ranging from 39 to 195. The scale demonstrated excellent reliability (Cronbach’s α = 0.958) in this study.

#### 2.2.3 Medical students’ digital literacy scale

The 25-item scale developed by Zhang and Wu (23) assessed six dimensions: information and data literacy, communication and collaboration, digital content creation, safety, problem-solving, and profession-related literacy. Items used a 5-point Likert scale (1 = strongly disagree to 5 = strongly agree), with total scores ranging from 25 to 125. The scale showed good reliability (Cronbach’s α = 0.93).

#### 2.2.4 General self-efficacy scale

The Chinese version of the General self-efficacy scale (GSES) (24), adapted from Schwarzer and Born (25), contains 10 items rated on a 4-point Likert scale (1 = completely incorrect to 4 = completely correct). Total scores range from 10 to 40. The scale demonstrated good reliability in this study (Cronbach’s α = 0.907).

### 2.3 Data collection

Electronic questionnaires were administered via a secure online platform. A three-level recruitment approach (university-hospital-department) was implemented. Researchers explained the study purpose, procedures, and confidentiality measures before obtaining informed consent. Questionnaires with logical inconsistencies, high response patterns (>90% identical responses), or unusually short completion times were excluded. Of 844 distributed questionnaires, 823 were valid (response rate: 97.50%).

### 2.4 Data analysis

Data were analyzed using SPSS 26.0 and the PROCESS macro (Model 4) (31). The analytical strategy proceeded in four stages to test the hypotheses:

Stage 1: Descriptive and preliminary analyses. Descriptive statistics (means, standard deviations, frequencies) were computed for all variables. Independent samples *t*-tests and one-way ANOVA were conducted to examine group differences in digital resilience across demographic and clinical characteristics, informing covariate selection for subsequent models.

Stage 2: Correlation analysis. Pearson correlation coefficients were calculated to examine bivariate relationships among digital literacy, self-efficacy, and digital resilience, providing preliminary evidence for hypothesized associations (H1, H2).

Stage 3: Hierarchical regression. Multiple linear regression was employed to assess the predictive effects of digital literacy and self-efficacy on digital resilience while controlling for significant demographic covariates. This step tested the direct effects specified in H1 and H2.

Stage 4: Mediation analysis. To test H3 regarding the mediating role of self-efficacy, Model 4 of the PROCESS macro was utilized with bootstrapping (5,000 resamples) to estimate 95% bias-corrected confidence intervals for indirect effects. Bootstrapping was preferred over the Sobel test due to its robustness to non-normal sampling distributions of indirect effects and its superior power for detecting mediation in medium-sized samples (26). Mediation was confirmed if the 95% CI for the indirect effect excluded zero. Statistical significance was set at *p* < 0.05.

## 3 Results

### 3.1 Demographic characteristics and digital resilience status

Participants had a mean digital resilience score of 138.95 ± 13.45 (Table 1). The highest scores were observed for “adaptation to digital environments” (4.19 ± 0.61) and “knowledge and skill acquisition” (3.89 ± 0.65), while lower scores were found for “digital risk perception” (3.03 ± 0.51), “cognitive coping strategies” (2.86 ± 0.60), and “digital stress management” (2.82 ± 0.51) (Table 1).

**Table 1:** Digital resilience scores among nursing interns (*n* = 823).

Dimension	Total score	Item mean score
Digital resilience	138.95 ± 13.45	3.56 ± 0.35
Digital risk perception	33.37 ± 5.61	3.03 ± 0.51
Cognitive coping strategies	17.16 ± 3.60	2.86 ± 0.60
Knowledge and skill acquisition	38.87 ± 6.51	3.89 ± 0.65
Digital stress management	14.15 ± 2.54	2.82 ± 0.51
Digital environment adaptation	29.35 ± 4.29	4.19 ± 0.61

### 3.2 Group differences in digital resilience

ANOVA and *t*-tests revealed significant differences in digital resilience scores based on gender, hometown location, internship duration, frequency of digital tool use, and participation in digital training (*p* < 0.05). No significant difference was found for educational level (*p* = 0.188) (Table 2).

**Table 2:** Factors associated with digital resilience scores.

Variable	*n*	Digital resilience score	*F/t*	*P*
Gender			−2.443	0.015
Male	182	136.81 ± 13.87		
Female	641	139.57 ± 13.29		
Level of education			1.319	0.188
Associate degree	285	139.82 ± 12.58		
Bachelor’s degree	538	138.51 ± 13.90		
Place of origin			−2.128	0.034
Rural	323	137.72 ± 13.53		
Urban	500	139.76 ± 13.37		
Internship duration			16.765	0.000
1–3 months	272	135.77 ± 13.35		
4–6 months	259	138.73 ± 12.78		
>6 months	292	142.20 ± 13.27		
Digital tool use frequency			9.443	0.000
Occasional (<3 times/week)	80	133.53 ± 10.96		
Sometimes (3–5 times/week)	260	137.85 ± 13.15		
Often (6–10 times/week)	315	139.28 ± 13.94		
Frequent (>10 times/week)	168	142.79 ± 13.06		
Participation in clinical digital skills training			17.200	0.000
Did not participate	247	135.75 ± 13.53		
Participated occasionally (1–2 times)	338	138.64 ± 12.48		
Participated regularly (≥3 times)	238	142.72 ± 13.82		

### 3.3 Correlation among digital resilience, digital literacy, and self-efficacy in nursing interns

Digital resilience was positively correlated with both digital literacy (*r* = 0.713, *p* < 0.01) and self-efficacy (*r* = 0.590, *p* < 0.01). Digital literacy and self-efficacy were also positively correlated (*r* = 0.668, *p* < 0.01) (Table 3).

**Table 3:** Correlations among digital resilience, digital literacy, and self-efficacy in nursing interns.

Item	Score	Total score of digital resilience	Total score of digital literacy	Total score of self-efficacy
Digital resilience total score	138.96 ± 13.46	1		
Digital literacy total score	67.18 ± 15.29	0.713^**^	1	
Self-efficacy total score	23.59 ± 4.34	0.590^**^	0.668^**^	1

^**^p < 0.01.

### 3.4 Hierarchical regression analysis of digital resilience among nursing interns

**Figure 2:**
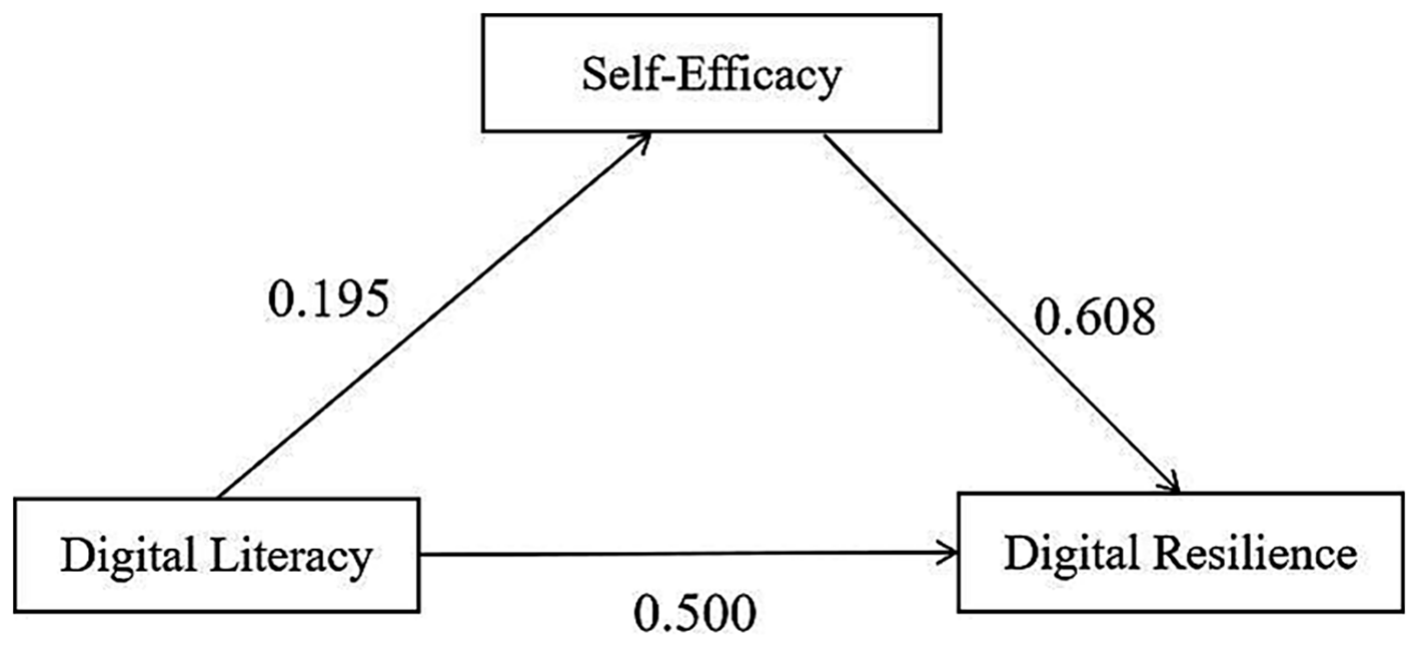
Mediation model of self-efficacy between digital literacy and digital resilience. Path values represent standardized coefficients.

Taking digital literacy as the independent variable and digital resilience as the dependent variable, with self-efficacy as the mediating variable, and incorporating the four statistically significant variables from Table 2 as covariates, Model 4 in the Process component was employed for multiple linear hierarchical regression analysis. The results are presented in Table 4, and the relationship model is illustrated in Figure 2.

**Table 4:** Multiple linear hierarchical regression analysis of the mediation model involving digital resilience, digital literacy, and self-efficacy.

Item	Step 1			Step 2			Step 3		
	β	*t*	*p*	β	*t*	*p*	β	*t*	*p*
Constant	–	31.400	0.000	–	11.802	0.000	–	27.247	0.000
Gender	0.092	4.136	0.000	0.093	3.654	0.000	0.073	3.370	0.001
Place of origin	−0.031	−1.377	0.169	−0.120	−4.647	0.000	−0.007	−0.334	0.738
Internship duration	0.172	7.730	0.000	0.013	0.516	0.606	0.169	7.809	0.000
Frequency of digital nursing tool usage	0.154	6.899	0.000	0.002	0.074	0.941	0.153	7.060	0.000
Participation in clinical digital skills training	0.165	7.399	0.000	−0.023	−0.910	0.363	0.169	7.796	0.000
Total digital literacy score	0.702	31.260	0.000	0.686	26.571	0.000	0.567	18.982	0.000
Total self-efficacy score							0.196	6.601	0.000
*R* ^2^	0.600			0.470			0.620		
Adjusted *R*^2^	0.597			0.466			0.617		
*F*-value	*F*(6,816) = 203.880, *p* = 0.000			*F*(6,816) = 120.752, *p* = 0.000			*F* (7,815) = 190.096, *p* = 0.000		

^*^p < 0.05, ^**^p < 0.01.

Regression results supported H1 and H2. In Step 1, digital literacy significantly predicted digital resilience (β = 0.702, *p* < 0.001). In Step 3, after controlling for self-efficacy, digital literacy remained significant (β = 0.567, *p* < 0.001), while self-efficacy also demonstrated significant prediction (β = 0.196, *p* < 0.001), supporting the partial mediation hypothesis (H3).

### 3.5 Testing the mediating effect of digital literacy and self-efficacy on digital resilience among nursing interns

The Bootstrap method was applied with 5,000 resamples to calculate 95% confidence intervals. The analysis of the mediating effect of self-efficacy between digital literacy and digital resilience is presented in Table 5. The bootstrapped mediation analysis confirmed H3. The indirect effect of digital literacy on digital resilience through self-efficacy was statistically significant (effect = 0.118, 95% CI [0.090, 0.177]), indicating that self-efficacy partially mediates the relationship. The proportion of mediation was 19.1%, suggesting that while self-efficacy is an important mechanism, digital literacy also exerts substantial direct effects on resilience.

**Table 5:** Analysis of the mediating effect of self-efficacy between digital literacy and digital resilience.

Item	Significance	Effect size	95% CI	SE	Proportion of mediating effect
Digital literacy total score → Self-efficacy total score → Digital resilience total score Indirect effect	Indirect effect	0.118	0.090 ~ 0.177	0.022	19.1%
Digital literacy total score → Digital resilience total score Direct effect	Direct effect	0.500	0.448 ~ 0.551	0.026	80.9%
Digital literacy total score → Digital resilience total score Total effect	Total effect	0.618	0.579 ~ 0.657	0.020	

## 4 Discussion

### 4.1 Analysis of digital resilience, digital literacy, and self-efficacy levels among nursing interns

The overall level of digital resilience among nursing interns was found to be moderately high. This finding is consistent with recent studies by Mo et al. (7) among Chinese clinical nurses and Son et al. (27) among nursing students in the AI era, who reported moderate to high resilience levels in digitally mediated healthcare contexts. The dimensions of “adapting to the digital environment” and “acquiring knowledge and skills” received relatively high scores, reflecting the effectiveness of current nursing curricula in developing technical competencies (2). While the scores for “perceiving digital risks,” “cognitive coping strategies,” and “overcoming digital stress” were comparatively lower. This pattern mirrors findings from Ang et al.’s (28) systematic review on digital training for resilience, which identified risk perception and stress management as consistently underdeveloped areas across healthcare education programs. The discrepancy between technical skills and adaptive capacities suggests that current training may overemphasize tool proficiency while neglecting the psychological and strategic components of resilience (29).

The digital literacy level of this cohort of interns was moderate, consistent with findings from studies on medical student populations (23). The improvement of digital literacy relies on systematic training and practical opportunities (9). In this study, interns who used digital nursing tools more frequently and participated in digital training more often had correspondingly higher digital literacy scores, suggesting that sustained digital practice and training are effective pathways to enhance digital literacy (13).

The self-efficacy score was at a moderate level, indicating that interns have a fair degree of confidence in facing challenges within digital environments, but there is still potential for strengthening. As an internal driver of individual behavior, the level of self-efficacy directly influences an individual’s persistence and adaptability when encountering difficulties with digital technologies (11). The results of this study show significant positive correlations between self-efficacy and both digital literacy and digital resilience, suggesting that enhancing self-efficacy could be a crucial entry point for improving digital adaptability (13).

### 4.2 Analysis of the mediating role of self-efficacy between digital literacy and digital resilience

This study found that self-efficacy plays a partial mediating role between digital literacy and digital resilience, accounting for 19.10% of the total effect. This indicates that digital literacy not only directly influences digital resilience but also indirectly enhances it by boosting an individual’s self-efficacy. Higher digital literacy makes interns more familiar with digital tools and information environments, thereby strengthening their sense of confidence and control in digital scenarios (10). This enhanced efficacy, in turn, encourages them to seek solutions and adjust coping strategies more proactively when facing digital challenges, ultimately improving their overall digital resilience (29). Therefore, it is recommended to systematically implement digital literacy education, integrating content on digital tool usage, information ethics, and data security into pre-internship training and clinical teaching systems. Concurrently, attention should be paid to fostering self-efficacy. Efficacy-enhancement strategies, such as situational simulations, peer learning, and reflective journals, should be incorporated into digital teaching and practical sessions to boost interns’ technical confidence and adaptive capabilities (14).

These findings suggest that in cultivating digital resilience among nursing interns, the focus should not be solely on skill training and knowledge transmission but also on building their psychological sense of efficacy. Educators and clinical mentors can help interns accumulate successful experiences in digital practice by setting progressive tasks, providing positive feedback, and encouraging independent exploration, thereby gradually establishing and reinforcing their digital self-efficacy (14).

### 4.3 Research limitations and future directions

While the sample for this study was drawn from multiple institutions, offering a degree of representativeness, several limitations must be acknowledged. First, all participants were from tertiary Grade A hospitals, which typically have more advanced digital infrastructure than primary or secondary care settings. This may limit generalizability to nursing interns in less technologically equipped environments, where digital resilience demands may differ (30).

Second, the cross-sectional design precludes causal inference. While the mediation model is theoretically grounded, the possibility of reverse causation—wherein resilient individuals seek out more digital experiences, thereby enhancing literacy and efficacy—cannot be excluded. Longitudinal designs tracking interns across their clinical rotations would better elucidate developmental trajectories.

Third, the reliance on self-report measures raises concerns about common method bias. Although the high internal consistencies and the specific pattern of correlations (e.g., stronger literacy-resilience than efficacy-resilience correlation) argue against severe bias, future studies should incorporate objective measures of digital skills (e.g., performance-based assessments) and behavioral indicators of resilience (e.g., help-seeking behaviors following technical failures).

Fourth, the study did not assess specific types of digital tools or training programs, limiting the ability to identify which competencies are most critical for resilience. Future research should employ a more granular approach, examining specific digital domains (e.g., electronic health records, telehealth platforms, AI-assisted decision tools) and their differential relationships with resilience outcomes.

## 5 Conclusion

This study provides novel evidence on the antecedents of digital resilience among nursing interns, a critical yet understudied population in the digital transformation of healthcare. Three main findings emerge: First, nursing interns demonstrate moderate digital resilience, with particular strengths in environmental adaptation and knowledge acquisition but relative weaknesses in risk perception and stress management. Second, both digital literacy and self-efficacy are significant positive predictors of digital resilience, confirming the importance of both competency-based and psychological approaches to resilience development. Third, and most notably, self-efficacy partially mediates the relationship between digital literacy and digital resilience, accounting for 19.1% of the total effect.

These findings underscore that digital resilience is not merely a technical attribute but a psychosocial capacity cultivated through the interplay of skills and confidence. For nursing education, the implications are clear: effective preparation for digital healthcare requires integrated curricula that develop technical competencies while simultaneously building psychological resources. By systematically enhancing digital literacy through mastery experiences and fostering self-efficacy through progressive challenges and reflective practice, educators can cultivate nursing interns capable of thriving amid the uncertainties and complexities of digital clinical environments. As healthcare systems worldwide accelerate their digital transformation, such resilience-focused education will be essential for ensuring both workforce sustainability and quality patient care.

## Data Availability

The original contributions presented in the study are included in the article/supplementary material, further inquiries can be directed to the corresponding author.
